# RELATIONSHIPS BETWEEN PERSONAL NEEDS AND THE ENVIRONMENT: A STUDY OF ABUSE AND SELF-NEGLECT

**DOI:** 10.1093/geroni/igz038.3463

**Published:** 2019-11-08

**Authors:** Minzhi Ye, Farida Ejaz, Miriam Rose

**Affiliations:** Benjamin Rose Institute on Aging, Cleveland, Ohio, United States

## Abstract

Estimates of self-neglect among older and/or disabled adults are much higher than the estimated 10% of older adults in the U.S. who experience physical or emotional abuse, neglect, and exploitation by others. It is not clear how the social environment affects these vulnerable adults who become self-neglecting. This study uses network analysis and a GIS approach to explore patterns of needs and environmental risks for adult healthcare patients who had risk factors for self-neglect. Sources of data included face-to-face interviews, self-neglect risk factors (e.g., depression, dependency in activities of daily living, etc.) from electronic medical records, and neighborhood information from census block data. More than two-thirds of the 480 study participants reported an average income of less than $1,360 monthly, and 89% self-identified as Hispanic or Latino. Using ArcGIS Pro, respondents’ geocoded addresses were matched to mapped neighborhood census information. The maps showed that most respondents live in Hispanic-dominated communities, in neighborhoods with crime rates above average and median household income of less than $49,066/year. These neighborhoods were probably resource-poor and had spatial inequalities. Using network analysis, the study found that the at-risk patients’ most frequently reported needs (e.g., food assistance/nutrition, functional limitations, social isolation) appear clustered together, demonstrating that people had multiple needs. The study findings suggest that practitioners and policymakers must not only provide a range of services to help disadvantaged groups, but also focus specifically on offering services in neighborhoods where low-income minority groups reside and there is a lack of community resources.

